# Supplementary material for: Inhibition of Carbonic Anhydrase 2 Overcomes Temozolomide Resistance in Glioblastoma Cells
Source: Int J Mol Sci. 2021 Dec 23;23(1):157. doi: 10.3390/ijms23010157 (PMC8745118; doi:10.3390/ijms23010157)
Supplement: Supplementary file 1 [file ijms-23-00157-s001.zip › ijms-1484897-supplementary.pdf]

Supplementary Materials

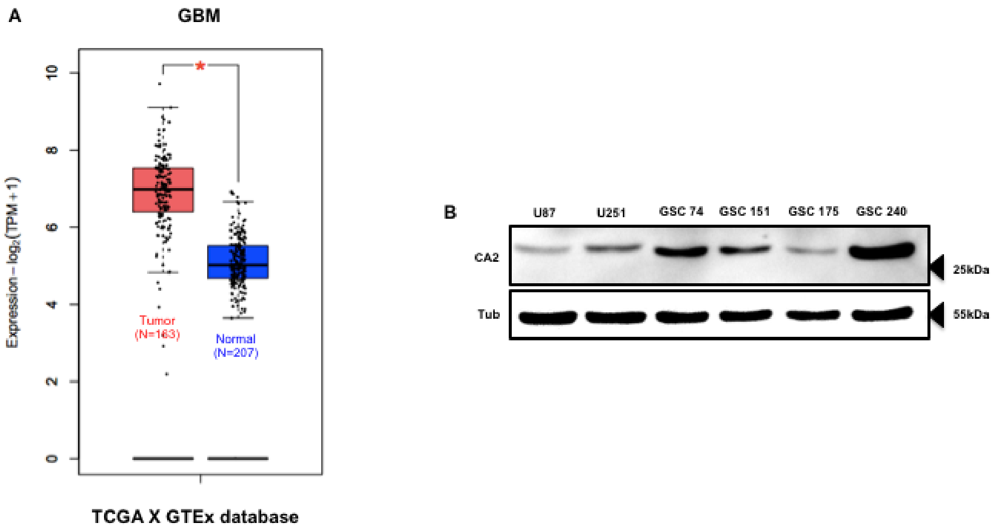

Figure S1. CA2 expression in GBM patients, GBM cell lines and GBM stem cells. (A) Expression status of the CA2 gene in GBM tissue compared to normal brain was analyzed from the TCGA and GTEx database. (B) The protein level of CA2 was increased in GSCs compared to U87 and U251 cells. Results were obtained from three independent experiments.

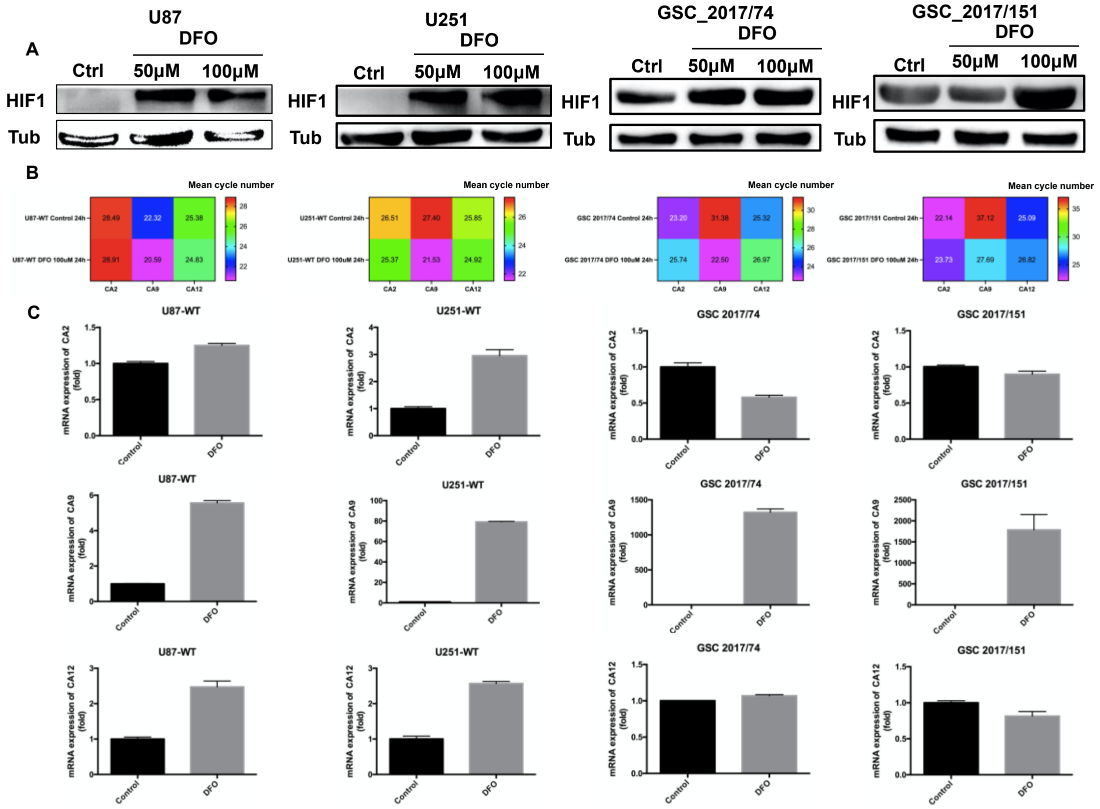

Figure S2. Hypoxia induced mRNA expression of GBM-related CA genes CA2, CA9, and CA12. (A) HIF protein expression detected by Western Blot in DFO-induced hypoxia in GBM cell lines (U87 and U251) and GBM stem cells (GSC_2017/74 and GSC_2017/151). (B) Mean cycle number values as showed by Heat-

Map representing absolute gene expression levels of CA2, CA9 and CA12 in control cells and DFO stimulate cells. (C) mRNA expression of GBM related carbonic anhydrase genes (CA2, CA9 and CA12) after DFO treatment in GBM cell lines and GBM stem cells were detected by RT-PCR (n=1).

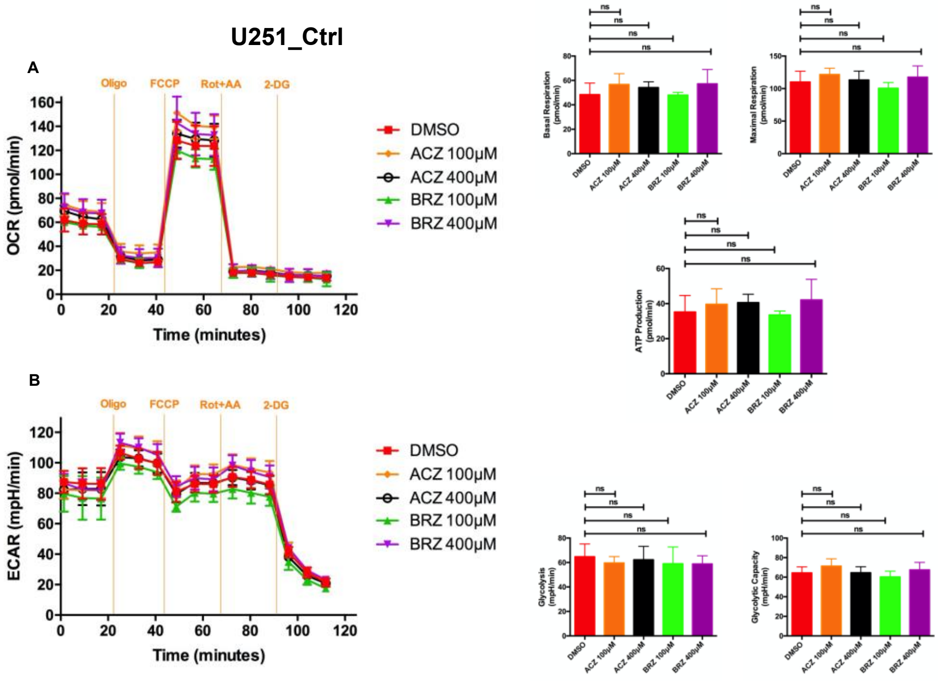

Figure S3. U251_Ctrl cells overall metabolism after ACZ and BRZ stimulation measured by seahorse XFe96 metabolic-flux analyzer. ACZ and BRZ did not change oxidative metabolism (A) and the level of glycolysis rate (B) in U251_Ctrl cell (n=5-6). All data are presented as mean ± SD, One-way ANOVA was used to analyze the data, * P <0.05; ** P <0.01; *** P <0.001, ns: not significant. Results were obtained from three independent experiments.

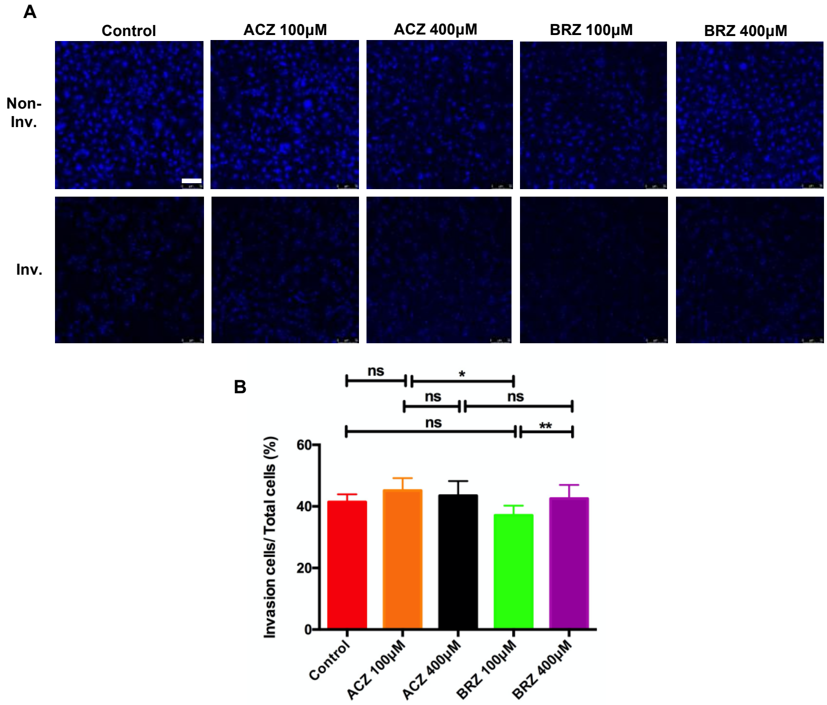

Figure S4. U251_Ctrl cells invasion after ACZ and BRZ treatment. (A) Invasion images of U251_Ctrl cells stained with nucleus after ACZ and BRZ treatment (scale bar: 50 μm). (B) Quantification of the proportion of invasive cells. ACZ and BRZ did not reduce the invasion of U251_Ctrl cells (n=6). Results were obtained from three independent experiments. Data are presented as mean ± SD, One-way ANOVA was used to analyze the data, * P <0.05; ** P <0.01; *** P <0.001, ns: not significant.

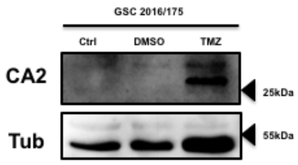

Figure S5. Consistent with qPCR result, the protein level CA2 increased in GSC_TMZ cell compared to GSC_Ctrl or GSC_DMSO cells (n=2).

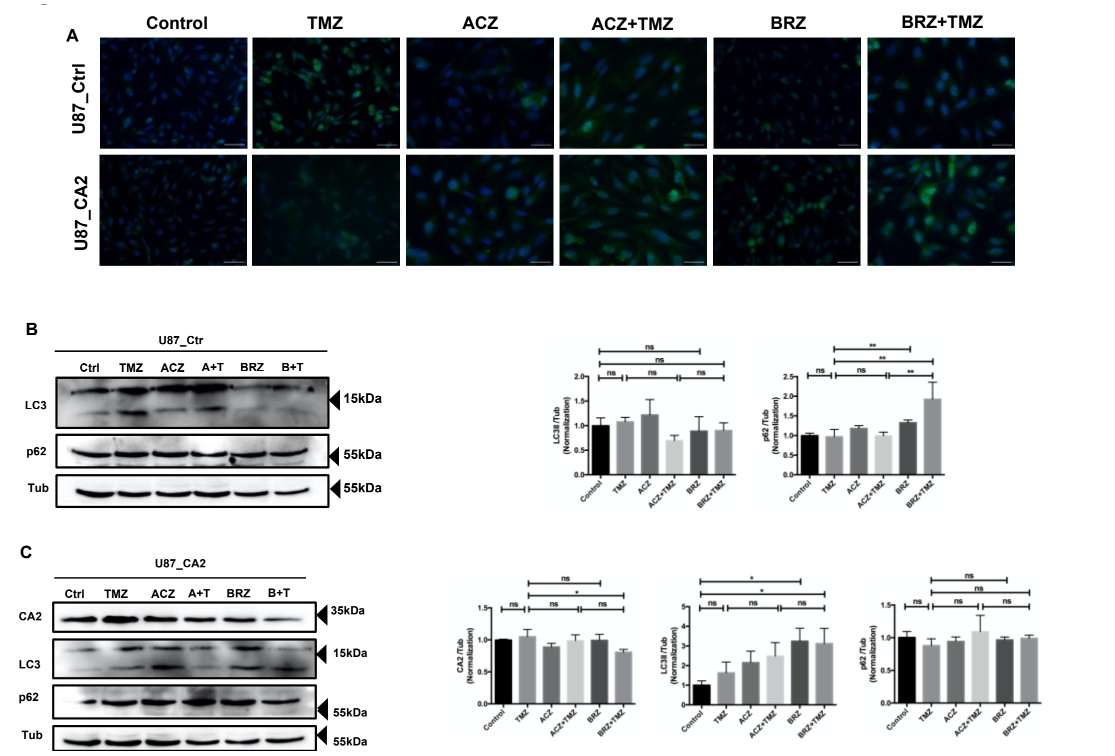

Figure S6. Combined administration of BRZ and TMZ enhances autophagy in U87_CA2 cells. (A) Autophagy marker LC3 ICF staining of U87_Ctrl and U87_CA2 cells after TMZ and ACZ/BRZ stimulation for 24h (scale bar: 50 μm). (B, C) Western blotting of autophagy-related proteins and CA2 protein in U87_Ctrl and U87_CA2 cells with the same treatment as in Fig 4B for 24h. TMZ plus BRZ did not increase the protein expression of LC3II in U87_Ctrl cells (B), but it increased in U87_CA2 cells (C). Results were obtained from three independent experiments. Data are presented as mean ± SEM, One-way ANOVA was used to analyze the data, * P <0.05; ** P <0.01; *** P <0.001, ns: not significant.

Table S1

Nr	Sex	Age	Survival (day)	Latency (day)	Tumor location	Tumor size (mm^3)	Histologic grade	MGMT status	EGFR v III	IDH R132H	P53	Ki76	Therapy
A	w	70	392	168	Right parietal	22*35*27	IV	+	-	-	++	up to 20%	Surgery +TMZ +Radiotherapy
							IV	+	-	-	+	10%	
B	m	67	336	130	Right occipital	30*60	IV	-	+	-	+	20%	Surgery +TMZ +Radiotherapy
							IV	-	+	-	+	20%	
C	w	77	unknown	104	Left parietal	31*26*23	IV	+	-	-	+	up to 10%	Surgery
							IV	+	-	-	+	up to 25%	
D	m	55	473	191	Left temporal	ø 20	IV	-	-	-	+	30-40%	Surgery +TMZ +Radiotherapy
							IV	-	-	-	+	30-40%	
E	m	61	627	296	Right temporal	34*25*23	IV	+	-	-	++	30%	Surgery +TMZ +Radiotherapy
							IV	+	-	-	++	30%	
F	w	63	unknown	385	Right parietal	32*29*23	IV	+	+	-	+	25%	Surgery +TMZ +Radiotherapy
							IV						
G	m	58	Alive	937	Left temporal	28*42*21	IV	+	-	-	-	30%	Surgery +TMZ +Radiotherapy
							IV	+	-	-	+	>10%	
H	m	62	unknown	281	Left occipital	41*61*35	IV	-	-	-	+	20%	Surgery +TMZ +Radiotherapy
							IV						
I	w	69	575	470	Right frontal	ø 15	IV	+	-	-	+	20%	Surgery +TMZ +Radiotherapy
							IV						
J	m	44	745	192	Right frontal	22*28*27	IV	+	-	-	+	25%	Surgery +TMZ +Radiotherapy
							IV	-	-	-	+	5%	

Table S1. Clinical information on GBM patients used for qPCR of iGBM and rGBM tissue (patient-

matched).

Table S2

Nr	Sex	Age	Survival (day)	Latency (day)	Tumor location	Histologic grade	MGMT status	EGFR v III	IDH R132H	P53	Ki76	Therapy
A	w	69	1277	1148	Right temporal and occipital	IV	+	-	-	++	up to 20%	Surgery +Radiotherapy
B	w	66	494	475	Left Master ganglia area	IV	+	-	-	+	10%	Surgery
C	m	55	1624	102	Left frontotemporal	IV	+	-	-	+	10%	Surgery +TMZ

Table S2. Clinical information on patients used for IHC stainings.

Table S3

Isoenzyme	Acetazolamide Ki (nM)	Brinzolamide Ki (nM)
hCA I[‡]	250	$4.5*10^4$
hCA II[‡]	12	3
hCA IV[‡]	74	$3.95*10^3$
hCA IX[ǂ]	25	37
hCA XII[ǂ]	5.7	3

Note: ‡ Full-length enzyme.
ǂ Catalytic domain.

Table S4

Isoenzyme	Acetazolamide	Brinzolamide
hCA I	+++	-
hCA II	+++	++++
hCA IV	+++	-
hCA IX	+++	-
hCA XII	+++	-

Note: "-" indicates no inhibitory effect.
"+" indicates inhibitory effect.
Increased inhibition is marked by a higher "+" designation.

Table S3 and S4. Inhibitory effects of ACZ and BRZ clinically used drugs against the carbonic anhydrase isoforms. Note that although hCA2 and hCA12 have identical Ki values, the inhibitory profile for hCA12 might be different for Brinzolamide, as the value Ki value in table S3 is derived from the catalytic domain of hCA12 only. In some investigations, Ki values for catalytic domains only and the corresponding full-length carbonic anhydrase can differ by a factor of 30.
